# Madelung Disease Associated With Bilateral Avascular Necrosis of the Femoral Head: A Case Report of a Rare Disorder

**DOI:** 10.7759/cureus.109590

**Published:** 2026-05-25

**Authors:** Ajay Kumar Metke, Sonali Kagne, David D Chandy

**Affiliations:** 1 Department of Endocrinology and Diabetes, Sir Harkisan Narottamdas (HN) Reliance Foundation Hospital and Research Center, Mumbai, IND

**Keywords:** adipose tissue accumulation, adipose tissue diseases, avascular necrosis of femur, benign lipomatosis, benign symmetric lipomatosis, fat deposition, madelung’s disease

## Abstract

Madelung disease (MD) is a rare disorder of adipose tissue overgrowth characterized by symmetrical and progressive accumulation of nonencapsulated adipose tissue in the subcutaneous layer, mainly in the neck, arms, and upper back. In some cases, involvement of the torso, mammary area, and lower limbs is also reported. Typically painless, fat masses can cause negative effects like dysphagia, dysphonia, breathing challenges, and reduced neck mobility. The majority of individuals affected are men who consume alcohol excessively. Patients often opt for treatment for MD due to its cosmetic implications and metabolic complications. The disease’s cause is not fully understood, making diagnosis and treatment challenging. Treatment is mainly surgical for cosmetic deformity or pressure symptoms.

## Introduction

Madelung disease (MD) is a rare disorder of adipose tissue metabolism characterized by abnormal, symmetrical, and progressive deposition of nonencapsulated subcutaneous adipose tissue predominantly involving the neck, upper limbs, and upper back. The estimated incidence is approximately one in 25,000 individuals, and the disease predominantly affects middle-aged men with a history of chronic alcohol abuse. Clinically, patients commonly present with painless, symmetrical fatty masses over the neck and shoulders, producing a characteristic “horse collar” or “buffalo hump” appearance. In some cases, involvement of the torso, mammary region, and lower limbs has also been reported.

The exact pathogenesis of MD remains unclear; however, mitochondrial dysfunction, defective adrenergic-mediated lipolysis, and abnormal brown adipose tissue proliferation have been implicated. Current or past alcohol abuse is considered the most common underlying risk factor and has been associated with several metabolic disturbances, liver disease, neuropathy, nephropathy, and malignancies [1,2].

Although typically benign, MD may cause cosmetic disfigurement, restricted neck mobility, dysphagia, dysphonia, and respiratory compromise due to progressive adipose tissue accumulation. Treatment is mainly surgical when indicated for cosmetic deformity or compressive symptoms.

Here, we present a case of a 45-year-old man with MD associated with bilateral avascular necrosis (AVN) of the femoral head. To our knowledge, this appears to be a rare and previously unreported coexistence. Whether this association represents a direct pathological link or reflects shared alcohol-related risk factors remains uncertain. Clinicians should be aware that patients with MD presenting with hip pain may warrant evaluation for AVN.

## Case presentation

A 45-year-old man presented with bilateral hip pain and was diagnosed with bilateral AVN of the femoral head. He first noticed 1-2 cm soft, painless swellings behind both ears, progressively extending to the entire retro- and preauricular area, the occipital area, the anterior neck, and the submental and submandibular areas over three years. Within a year, the distribution of fat rapidly expanded to symmetrically involve shoulders, arms, the upper chest, and the back. He did not have any pressure symptoms; however, swellings were cosmetically disfiguring (Figures 1A, 1B).

**Figure 1:**
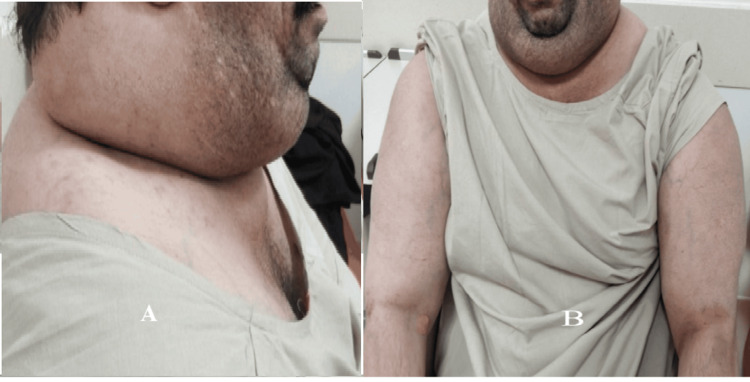
A 45-year-old man with Madelung disease. (A) Side view. (B) Front view

The patient had a history of chronic alcohol consumption for approximately 30 years, consuming an estimated 30 units/day (or 750 mL/day of country liquor). He had abstained from alcohol for the last 10 years before the presentation. He was also a chronic smoker with approximately a 20-30 pack-year history. Except for hyperuricemia, for which he was taking febuxostat, he did not have any other comorbidities. There was no history of corticosteroid use (oral, inhaled, intra-articular, or topical), trauma, caisson disease, sickle cell disease/trait, or known coagulopathy. His family history was negative for a similar complaint.

The patient reported progressive bilateral hip pain for approximately five months before presentation. Pain was aggravated by walking and weight-bearing activities and was associated with difficulty in ambulation and restriction of hip movements. No history of trauma was present.

On evaluation, laboratory investigations revealed the following: hemoglobin: 11.5 g/dL (13-17 g/dL); total leukocyte count: 13.41 (4-10 x 10 ^9^/L); platelet count: 195 (150-410 x 10^3^/L); serum uric acid: 7.3 mg/dL (3.4-7.0 mg/dL); aspartate aminotransferase: 28 U/L (<40 U/L); alanine aminotransferase: 27 U/L (<41 U/L); alkaline phosphatase: 126 U/L (40-129 U/L); serum bilirubin: 0.5 mg/dL (<1.2 mg/dL); random blood glucose: 85 mg/dL (<200 mg/dL). Viral hepatitis markers were negative. Evaluation revealed positive antimitochondrial antibodies suggestive of autoimmune etiology for his liver disease.

Abdominal ultrasonography revealed coarse hepatic parenchymal echotexture with a liver span of 13.8 cm and associated cholelithiasis. A CT chest revealed extensive subcutaneous fat deposition without mediastinal involvement. CT chest coronal view in a 45-year-old man with MD, showing excessive symmetrical subcutaneous fat deposition in arms, shoulders, and neck, is shown in Figures 2A, 2B. Figure 3 shows an axial CT chest view of a 45-year-old man with MD, demonstrating excessive symmetric subcutaneous fat deposition around the neck.

**Figure 2:**
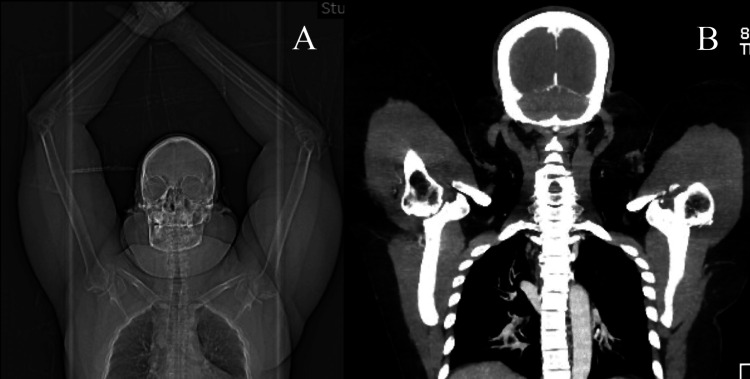
Diffuse symmetrical lipomatosis involving the bilateral neck, chest wall, and arms (A) Topogram acquired before CT scan. (B) Soft-tissue-window-reconstructed CT image

**Figure 3:**
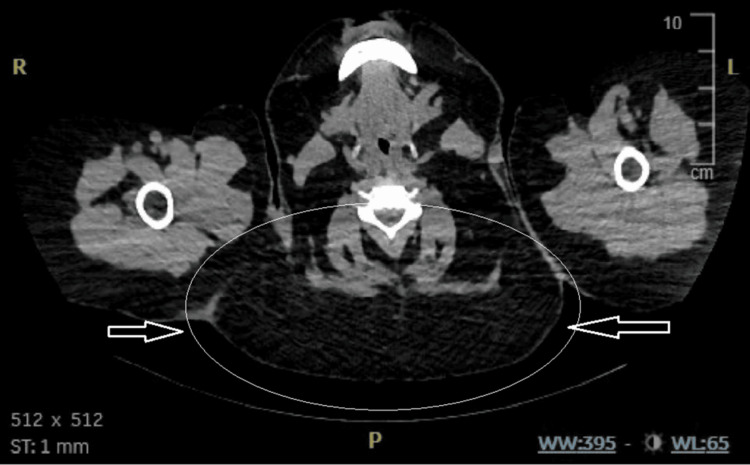
CT chest axial view in a 45-year-old man with Madelung disease, showing excessive symmetrical subcutaneous fat deposition around neck

An MRI of the pelvis with both hips revealed AVN of the femur bilaterally. The MRI coronal view demonstrates bilateral femoral head AVN corresponding to Ficat and Arlet Stage IV disease [3], with severe secondary osteoarthritis changes and mild joint effusion (Figures 4A, 4B). He underwent total hip replacement on both sides. At the six-month follow-up, he reported significant improvement in pain and mobility with no postoperative complications. The lipomatous lesions remained stable in size during follow-up.

**Figure 4:**
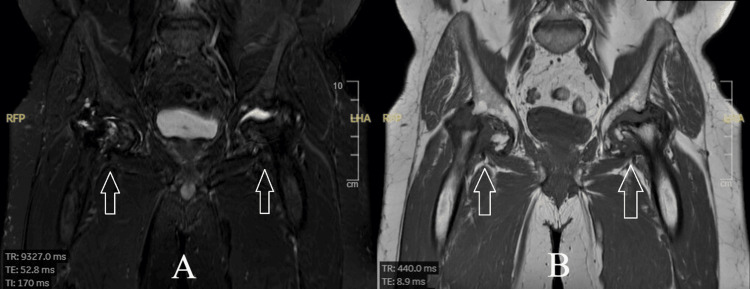
Images displaying bilateral severe osteoarthritic changes in the hip joints with associated joint effusions and flattening of the femoral heads with articular surface irregularity, bone resorption, and remodeling suggestive of Stage IV avascular necrosis (A) MRI coronal STIR sequence. (B) MRI coronal T1 non-fat-suppressed sequence

## Discussion

Madelung’s disease (MD) is a rare disorder of lipid metabolism, reported to occur with an incidence of roughly one in 25,000 individuals [4,5]. It is also known as cephalothoracic lipodystrophy, Launois-Bensaude syndrome, and multiple symmetric lipomatosis. It is sporadic in nature and mostly benign. MD occurs frequently in middle-aged men, with a reported male-to-female ratio ranging from 15:1 to 30:1. It is common in Mediterranean countries and extremely rare in Asians [4,5].

Classification

Depending on the distribution of fat across different body areas, MD is divided mainly into three phenotypes, as per a recent study of a German patient cohort (n = 45) by Schiltz et al. [4,6]. According to this study, phenotype I involves upper body parts (neck, shoulder girdle, upper arms, and trunk). Phenotype II involves lower body parts (hips and upper legs). Whereas in phenotype III, adipose tissue is diffusely distributed throughout the body, while the head, forearms, and lower extremities remain unaffected (Figure 5). The appearance resembles an obesity type of fat distribution. Based on predominant involvement of the neck, shoulders, upper arms, and upper trunk, the patient was categorized as phenotype Type Ib according to the Schiltz classification.

**Figure 5:**
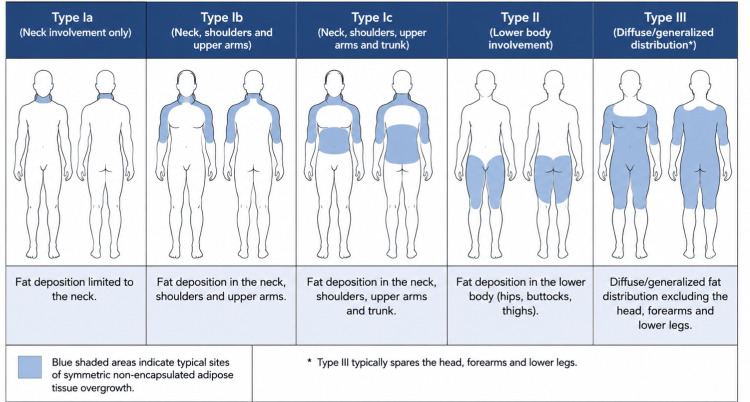
Phenotypic classification of Madelung disease Image credit: This is an original image designed by the authors based on the work by Schiltz et al. and related published classification systems [4,6]

In a recent analysis by Li et al., it was found that approximately 87% had a current or past history of alcohol abuse, with the average duration being 24 years [7]. At the same time, approximately 77% of MD patients had a history of smoking, with the average duration of nicotine abuse being 21 years [7]. Our patient had a long-standing history of both risk factors. Although he had stopped alcohol intake completely 10 years ago, his disease continued to progress. It is speculated that long-term alcohol consumption may be a risk factor for triggering MD [2,7]. Alcohol cessation may result in a slight regression of lipoma depots, but it cannot reverse them completely. This fact is consistent with the conclusions from many other studies [2,7].

Patients with MD exhibit adipose tissue distribution and adipocyte characteristics comparable to those of infant brown adipose tissue, supporting a potential role for brown fat dysfunction in disease pathogenesis [8]. In these patients, the adipogenesis is due to the appearance of new adipocytes rather than an increase in the size of existing ones [9].

Adipogenesis has been associated with abnormalities of the respiratory chain and structural alterations of mitochondrial DNA, including deletions and mutations [5,10]. Alcohol intake has been suggested to contribute to disease pathogenesis by inducing alterations in the number and functional properties of β-adrenergic receptors on adipocytes [2,5]. Altered enzyme and membrane receptor activity contribute to diminished adrenergic-mediated lipolysis [2]. Hence, it is likely that alcohol abuse is the predisposing or aggravating factor for abnormal accumulation of adipose tissue affecting multiple body sites in MD [2]. Another possible risk factor is nicotine abuse [11].

MD is found to be associated with multiple comorbidities involving endocrine, gastrointestinal, neurological, urogenital, and cardiovascular systems. Liver involvement is also common. Neuropathies are common among neurological involvements, probably secondary to alcohol abuse [5,7]. Our patient had bilateral AVN of the hip (AVN), hyperuricemia, hyperlipidemia, and liver shows coarse parenchymal echotexture; the span is 13.8 cm with positive antimitochondrial antibodies. The exact cause of this association is not known, but underlying alcohol abuse can explain both MD and AVN.

Although the coexistence of MD and bilateral AVN in our patient may be coincidental due to shared alcohol exposure, a possible common pathophysiological pathway may exist. Chronic alcohol intake has been associated with mitochondrial dysfunction, impaired adrenergic-mediated lipolysis, and abnormal adipocyte proliferation in MD [2,5,8-10]. Similarly, alcohol-related lipid metabolism abnormalities and mitochondrial injury have been implicated in intraosseous fat hypertrophy and compromised femoral head microcirculation, leading to AVN. This raises the possibility that alcohol-induced metabolic and mitochondrial alterations may contribute to pathological adipose accumulation in both subcutaneous and intraosseous compartments.

Alcohol use has been implicated as a contributing factor in the development of hip AVN, with studies reporting its presence in approximately one-third of cases [12]. Excessive alcohol consumption related to AVN of the femoral head is the result of excess lipid formation. Several disorders may mimic MD, including obesity; endocrine conditions such as Cushing syndrome; benign adipose tumors; and lipomatosis associated with HIV infection [5,9].

The disease demonstrates a progressive pattern of adipose tissue accumulation, characterized by episodic rapid enlargement alternating with periods of slow progression or prolonged stability, and no spontaneous regression has been documented. Further investigation of the maturation time of these lesions is necessary for treatment.

Indications for intervention include cosmetically significant deformity, impaired mobility, and symptoms of local compression, such as dyspnea and dysphagia. Treatment usually consists of surgical removal of fat from the affected areas [5]. Lipectomy and liposuction have been reported as surgical methods.

## Conclusions

MD is a rare disorder of adipose tissue metabolism predominantly affecting middle-aged men with chronic alcohol exposure. Alcohol abuse remains the most common underlying risk factor, and disease progression may continue despite prolonged abstinence. Proposed mechanisms include mitochondrial dysfunction and impaired adrenergic-mediated lipolysis resulting in abnormal adipose tissue accumulation. MD has been associated with multiple metabolic, hepatic, neurological, and endocrine comorbidities.

We report a rare coexistence of MD with bilateral AVN of the femoral head in a patient with longstanding alcohol exposure. Although a direct causal relationship cannot be established, shared alcohol-related metabolic and mitochondrial dysfunction may represent a possible common pathogenic mechanism. Clinicians should consider AVN in patients with MD presenting with hip pain or gait limitation. Surgical management remains the primary treatment modality when cosmetic deformity, functional limitation, or compressive symptoms are present.
